# Differential Regulation of Horizontally Acquired and Core Genome Genes by the Bacterial Modulator H-NS

**DOI:** 10.1371/journal.pgen.1000513

**Published:** 2009-06-12

**Authors:** Rosa C. Baños, Aitziber Vivero, Sonia Aznar, Jesús García, Miquel Pons, Cristina Madrid, Antonio Juárez

**Affiliations:** 1 Institut de Bioenginyeria de Catalunya (IBEC), Parc Científic de Barcelona, Barcelona, Spain; 2 Departament de Microbiologia, Facultat de Biologia, Universitat de Barcelona, Barcelona, Spain; 3 Institute for Research in Biomedicine (IRB Barcelona), Parc Científic de Barcelona, Barcelona, Spain; 4 Departament de Química Orgànica, Universitat de Barcelona, Barcelona, Spain; Universidad de Sevilla, Spain

## Abstract

Horizontal acquisition of DNA by bacteria dramatically increases genetic diversity and hence successful bacterial colonization of several niches, including the human host. A relevant issue is how this newly acquired DNA interacts and integrates in the regulatory networks of the bacterial cell. The global modulator H-NS targets both core genome and HGT genes and silences gene expression in response to external stimuli such as osmolarity and temperature. Here we provide evidence that H-NS discriminates and differentially modulates core and HGT DNA. As an example of this, plasmid R27-encoded H-NS protein has evolved to selectively silence HGT genes and does not interfere with core genome regulation. In turn, differential regulation of both gene lineages by resident chromosomal H-NS requires a helper protein: the Hha protein. Tight silencing of HGT DNA is accomplished by H-NS-Hha complexes. In contrast, core genes are modulated by H-NS homoligomers. Remarkably, the presence of Hha-like proteins is restricted to the *Enterobacteriaceae*. In addition, conjugative plasmids encoding H-NS variants have hitherto been isolated only from members of the family. Thus, the H-NS system in enteric bacteria presents unique evolutionary features. The capacity to selectively discriminate between core and HGT DNA may help to maintain horizontally transmitted DNA in silent form and may give these bacteria a competitive advantage in adapting to new environments, including host colonization.

## Author Summary

Acquisition of DNA by horizontal gene transfer (HGT) significantly increases bacterial genetic variability. Relevant issues are the mechanisms that bacterial cells have evolved to efficiently integrate the newly acquired DNA into the host cell regulatory machinery. In Gram negative cells, the nucleoid associated protein H-NS has been shown to bind AT-rich sequences of HGT DNA and silence unwanted expression of these genes. This has led to consider H-NS as a “genome sentinel.” Nevertheless, this proposed role must be compatible with its role modulating core genome genes. Weak expression of recently transferred genes must be coordinated with proper expression levels of housekeeping genes. In this paper, we describe a strategy that enteric bacteria have developed to differentially modulate HGT and core genome genes. Two independent lines of experimental evidence suggest that the H-NS system of enteric bacteria may have evolved to discriminate between core genome and HGT DNA. The plasmid R27-encoded H-NS protein selectively modulates HGT genes. This avoids plasmid-encoded H-NS interfering with modulation of core functions. We also show that, for efficient silencing of HGT genes, resident chromosomal H-NS recruits the Hha protein and forms heteromeric complexes with DNA. In contrast, housekeeping genes are modulated by H-NS alone.

## Introduction

Acquisition of DNA by horizontal gene transfer (HGT) is a crucial mechanism by which bacteria increase genetic variability. Among others, functions that enable bacterial cells to cause disease (virulence factors) as well as to overcome the effect of antimicrobial drugs are often encoded in HGT DNA (i.e., bacterial plasmids or genomic islands). While HGT DNA may provide a potential advantage in host colonization, the incorporation of foreign DNA may constitute a potential perturbation for the regulation of the core genome, resulting in a significant fitness cost. An efficient mechanism that enables the bacterial cell to control the expression of foreign DNA is exemplified by the H-NS protein [for a review see 1]. H-NS belongs to the superfamily of bacterial nucleoid-associated proteins and is involved in the adaptative response of bacterial cells to changes in environmental factors such as temperature or osmolarity. The regulatory region of H-NS-modulated genes usually contains two separated target sequences, which have often been characterized by being AT-rich curved DNA stretches [2]. Interaction of H-NS molecules with their target sequences results in protein oligomerization and the generation of a DNA loop. When this nucleoprotein complex is formed, transcription is switched off [2]–[4]. Silencing is relieved when changes in physicochemical parameters (i.e., temperature) affect either DNA properties or the capacity of H-NS to oligomerize [2],[5],[6]. In some instances, H-NS-mediated silencing requires the participation of proteins of the Hha/YmoA family [for a review see 7]. Hha-like proteins have been identified on the basis of their role in modulating several virulence determinants [8]–[11]. Their molecular mass is about half of that of H-NS-like proteins. They show structural mimicry to the H-NS oligomerization domain, bind to H-NS and appear to comodulate the expression of several genes with this latter protein [7].

H-NS targets both core genome and HGT genes [12],[13] and provides an efficient mechanism that enables bacterial cells to control the expression of foreign DNA. Mapping of H-NS binding sites on the *Salmonella enterica* serovar Typhimurium chromosome by a ChlP on chip approach showed that H-NS binds preferentially to AT-rich HGT DNA [13],[14]. This finding has been interpreted as H-NS playing a relevant role in the silencing of unwanted expression of these sequences and has led to the proposal of a predominant role of the H-NS protein as a genome sentinel [15].

Several conjugative plasmids, such as those of the IncH1 group, also encode plasmidic forms of H-NS and Hha. IncH1 plasmids are common in the causal agent of typhoid fever, *Salmonella enterica* subsp. *enterica* serovar Typhimurium, and are associated with the multi-drug resistance (MDR) phenotype that some isolates exhibit [16]. A well-characterized representative of this incompatibility group is plasmid R27 [17]. This plasmid was isolated from *Salmonella enterica* serovar Typhimurium in the 1960s and since then has been detected in several *S.* Typhi outbreaks. R27 is 180 kbp in length, confers tetracycline resistance and shows a temperature-dependent conjugative phenotype. R27 encodes single copies of *hns* and *hha* genes (ORFs 164 and 182 respectively). Both chromosomal- and plasmid-encoded H-NS and Hha proteins interact to modulate R27 temperature-dependent conjugative transfer [18]: either plasmid- or chromosomally-encoded H-NS and Hha proteins can repress conjugative transfer at high temperature. The role of Sfh, an H-NS-like protein encoded by plasmid pSf-R27, 99.7% identical to R27, has been also addressed [19]–[21]. In an elegant series of experiments, it was shown that Sfh provides a stealth function that allows the plasmid to be transmitted to new bacterial cells without reducing fitness [22]. Plasmid-encoded H-NS would prevent the depletion of resident H-NS by AT-rich HGT sequences, for which H-NS shows a strong preference.

To date, plasmid- and chromosome-encoded forms of H-NS proteins have been assumed to be functionally equivalent [18],[22]. Here we provide evidence that plasmid-encoded H-NS-like proteins have evolved to selectively target HGT and not core genome DNA. We also show that chromosomally-encoded H-NS targets both HGT and core genomic DNA, but differentially modulates them by using Hha-like proteins to specifically silence HGT genes. Altogether, our results suggest that in enteric bacteria the H-NS modulator may have evolved to discriminate between vertically and horizontally inherited DNA sequences, efficiently silencing the latter. This feature could provide a fitness advantage by allowing the presence of a large number of silent virulence genes to be available, without interfering with the regulation of the bacterial core genome.

## Results

### Acquisition of R27 plasmid by an *hns* mutant from *S.* Typhimurium strain SV5015 restores wt expression of only a subset of H-NS-sensitive genes

**Figure 1:**
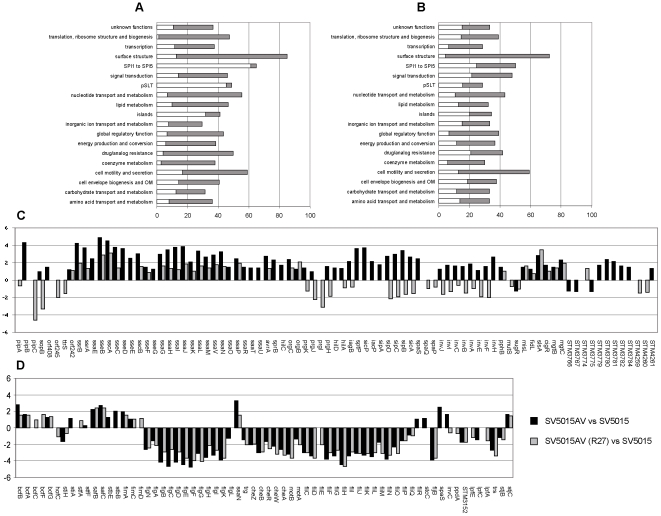
Effect of the presence of R27 in the transcriptome of an *hns* mutant strain. Changes in expression of several gene groups in the *hns* mutant strain (SV5015AV) and in the *hns* strain harbouring R27 plasmid (SV5015AV(R27)) with respect to the wt strain (SV5015). (A,B) Percentage of genes belonging to each group that show altered expression in strain SV5015AV (A) and SV5015AV (R27) (B) with respect to the wt strain. Grey bars indicate the proportion of down-regulated genes (M<0) and open bars indicate the proportion of up-regulated genes (M>0). M is the fold change log_2_ ratio. (C,D) M values of individual genes in the functional categories of pathogenicity islands SPI-1 to SPI-5 (C) and cell motility and secretion (D).

Analysis of the contribution of chromosomal- and R27-encoded H-NS proteins in silencing the functions required for plasmid conjugation suggested that these proteins are functionally interchangeable [18]. We further extended these studies by assessing the impact of plasmid R27 on the transcriptome of a chromosomal *Salmonella hns* mutant. For this purpose, we compared the gene expression patterns of wt and *hns* mutant from *S.* Typhimurium SV5015 (strain SV5015AV), the latter in the presence and in the absence of plasmid R27 (Figure 1A and 1B, Table S1 and Table S2). As expected and as previously reported [12], the *hns* mutation resulted in an altered expression of a significant number of genes. According to the hypothesis of functional equivalence of chromosomal and plasmidic H-NS, it was expected that expression of a functional H-NS protein encoded by plasmid R27 (H-NS_R27_) in *hns* cells would result in the restoration of the wt expression pattern. Unexpectedly, the transcriptomic analysis of strain SV5015AV (R27) showed that the H-NS_R27_ protein has the capacity to compensate the effect of the *hns* mutation only for a subset of genes. Overexpression in the *hns* mutant was compensated by the presence of R27 in 61% of the genes (Table S1). Remarkably, H-NS_R27_-sensitive genes were not randomly distributed along the *S.* Typhimurium SV5015 chromosome, but predominantly mapped in AT-rich sequences of *Salmonella* pathogenic islands (SPIs 1 to 5) and pSLT plasmid (Figure 1A and 1B). As an example, Figure 1C and 1D shows the effect of R27 on the expression patterns of genes belonging to pathogenicity islands (HGT genes) and cell motility and secretion (housekeeping genes) functional groups. Most of the genes encoding proteins that play key roles as global modulators of gene expression and have been reported to be sensitive to H-NS modulation (i.e., *psiF*, *dps*, *himA*, *stpA*, *rcsA*, and *hha*) were not sensitive to H-NS_R27_ modulation in *hns* cells (see Table S1 and Table S2).

### *In vivo* and *in vitro* analysis of H-NS_R27_ interaction with individual HGT or core genome promoters

**Figure 2:**
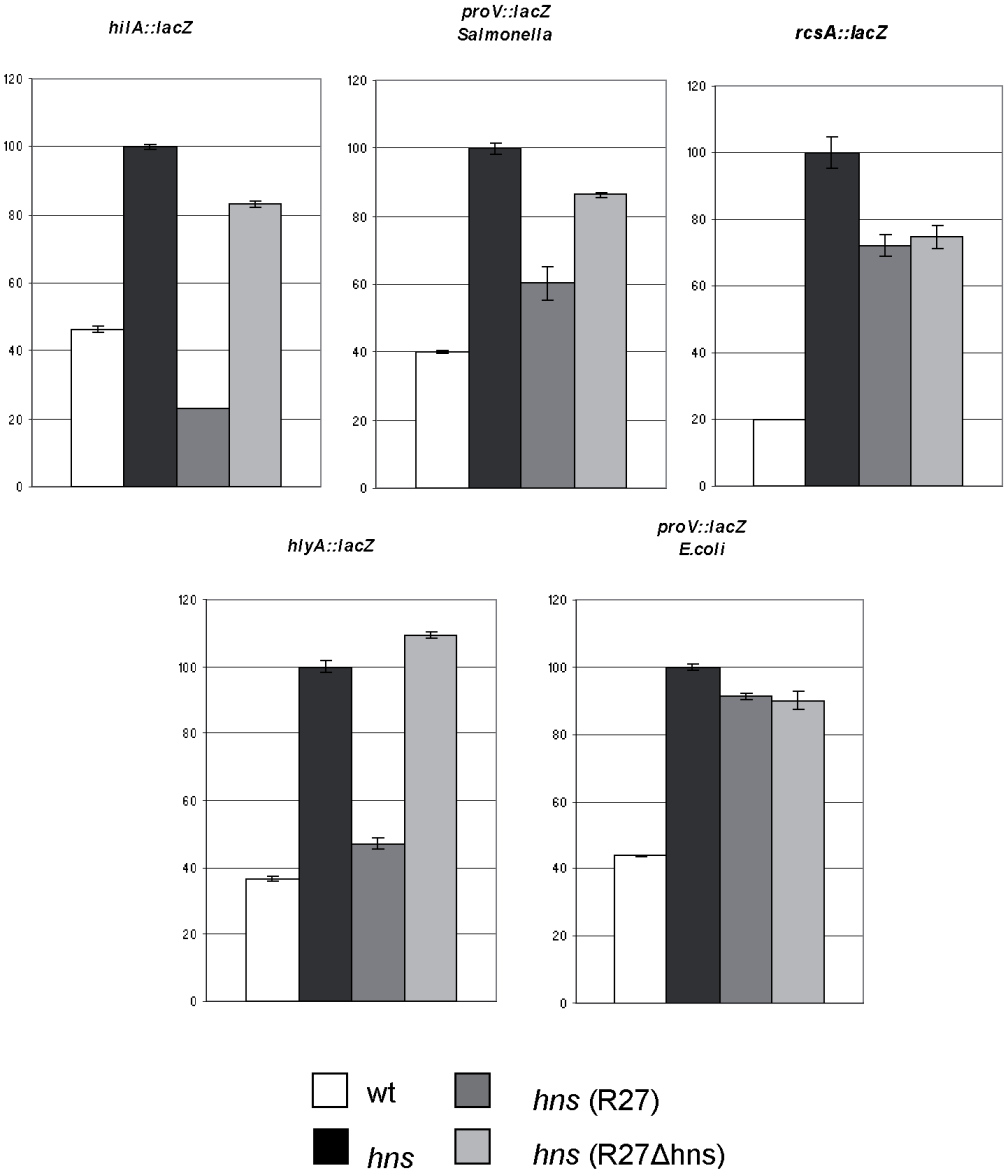
H-NS and H-NS_R27_-depending expression of selected genes. Expression of β-galactosidase from *lac* fusions to *hilA*, *proV* (*Salmonella* or *E. coli*), *rcsA*, and *hlyA* genes in wt, *hns*, *hns* (R27), and *hns* (R27Δ*hns*) strains. Bars represent percentage of activity of each strain with respect to the activity of *hns* strain.

To further analyze the capacity of chromosomal H-NS and of H-NS_R27_ to modulate H-NS-sensitive promoters in enteric bacteria, we tested their in *vivo* effect on single promoters, mapping either in the core genome or in HGT DNA of *S.* Typhimurium and *E. coli*. We selected *rcsA* and *proV* as representative core genome promoters. The *rcsA* gene encodes the colanic acid capsular biosynthesis activation protein A. *hns* mutants show a mucoid phenotype as a result of the derepression of colanic acid expression [23]. In the transcriptomic analysis reported above, *rcsA* was insensitive to H-NS_R27_ modulation. The *proVWX* operon includes the gene encoding the glycine-betaine transporter, and is one of the best characterized examples of an H-NS modulated promoter. Under non-permissive conditions (low osmolarity), H-NS represses its expression. Upon osmotic up-shift, its expression is increased up to 200-fold [24]. The *proVWX* operon is present in the genome of both *E. coli* and *Salmonella*, and here we studied both promoters. As examples of promoters mapping in HGT DNA, we selected *hilA*, which controls the expression of the master regulator of the *Salmonella* pathogenicity island 1 (SPI1) [25], sensitive to H-NS_R27_ modulation in our transcriptomic study (see Figure 1), and the *E. coli hly* promoter, which regulates transcription of the operon encoding the toxin α-hemolysin and that has been shown to be modulated by H-NS and Hha [5]. Reporter fusions *rcsA::lacZ*, *proV::lacZ hilA::lacZ* and *hlyA::lacZ* were constructed and β-galactosidase expression was evaluated in wt, *hns*, *hns* (R27) and *hns* (R27Δ*hns*) cells. Deregulated expression of *S.* Typhimurium *hilA* and *E. coli hly* promoters in *hns* mutants was fully compensated by H-NS_R27_ (Figure 2). In contrast, H-NS_R27_ only partially compensated the lack of chromosomal H-NS in *proV* expression in *Salmonella* strain SV5015AV, and failed to rescue the effect of the *hns* mutation in *proV* expression in *E. coli* strain 5K*hns*. As expected from the transcriptomic data, H-NS_R27_ failed to restore *rcsA* wt repression in *hns* cells. To show that H-NS core genome regulation is Hha-independent, we also tested *proV::lacZ* fusion in an *hha* mutant. The data obtained confirmed that *proV* expression is not affected in this mutant (Figure S1).

**Figure 3:**
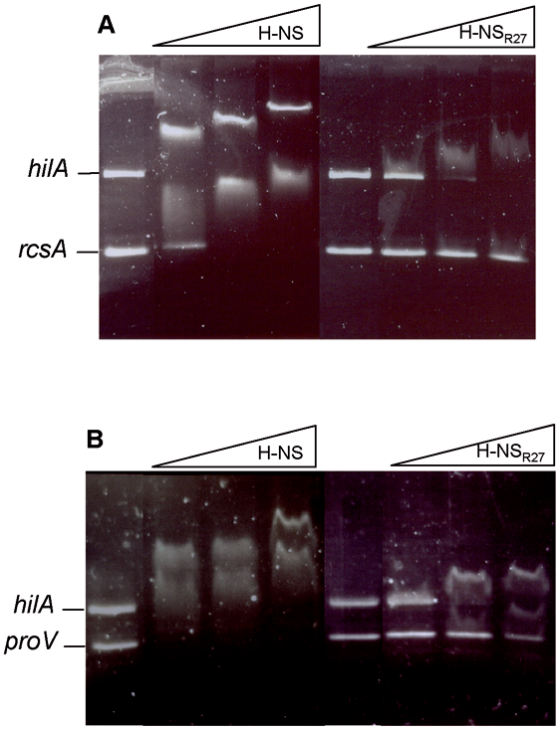
Differential affinity of H-NS and H-NS_R27_ to *hilA*, *proV*, and *rcsA* gene promoters. Competitive band shift assays showing differential affinity of H-NS_R27_ to the *hilA* and *rcsA* promoters (A) and to the *hilA* and *proV* promoters (B). H-NS and H-NSR_27_ purified proteins (1, 2, and 4 µM) were incubated with the mixture of both DNA fragments.

To complete these *in vivo* data, we also tested whether, *in vitro*, H-NS and H-NS_R27_ proteins show a differential affinity for specific promoter regions. The chromosomal H-NS protein showed a similar affinity for the DNA fragments containing the different H-NS-sensitive promoters, independently of their chromosomal or HGT lineage. In contrast, H-NS_R27_ showed higher affinity for the *hilA* regulatory region than for the *proV* or *rcsA* ones (Figure 3).

Given that several H-NS-sensitive genes are also modulated by the Hha protein [26], we tested whether H-NS_R27_ is strictly dependent on the Hha protein to modulate gene expression. We assayed expression of the *hilA::lacZ* fusion in a mutant strain lacking both chromosomal H-NS and Hha and housing a plasmid also deficient in plasmidic Hha (strain SV5015 HAV (R27Δ*hha*)). This plasmid still had the capacity to complement the *hns* mutation (data not shown), thus evidencing that H-NS_R27_ is functional in the absence of Hha.

### The set of *S.* Typhimurium genes that are sensitive to H-NS_R27_ significantly overlaps with those silenced by an H-NS/Hha complex

On the basis of the sensitivity of several genes to H-NS_R27_, the *Salmonella* H-NS regulon can be divided in two genetic compartments that can be tentatively associated with HGT and core genes. At this stage we considered it relevant to address whether these compartments could also be distinguished and differentially regulated by the resident chromosomal H-NS regulatory system. It was recently shown that the Hha modulator and/or its paralogue protein YdgT modulate a set of genes that largely map in AT-rich sequences of the *Salmonella* genome and that overlaps with the set of H-NS-regulated genes that map in genomic islands [26]. We hypothesized that the set of SV5015 genes sensitive to H-NS_R27_ are similar to the set of genes that are co-regulated by H-NS/Hha proteins. To test this hypothesis, we first performed global transcriptomic studies to determine H-NS and Hha-dependent modulation of the *Salmonella* genome in a range of conditions of osmolarity and temperature. Gene expression patterns of strains SV050515, SV5015AV (*hns*) and SV5015HY (*hha ydgT*) grown either in low and high osmolarity LB medium, and either at 25 or 37°C in conventional LB medium, were compared (Table S3). *hha ydgT*-mediated gene deregulation in LB medium at 37°C [26] was observed under the osmolarity and temperature conditions tested. Remarkably, most of the genes deregulated in the *hha ydgT* mutant were also deregulated in the *hns* mutant: a total of 162 genes were overexpressed both in *hha ydgT* and *hns* mutants, and most of them were overexpressed under several growth conditions (Table S3). Although the extent of overexpresssion differed, repression of a set of genes by H-NS/Hha at low and high temperature and at low and high osmolarity indicates that the H-NS/Hha complex efficiently silences their expression under a wide range of non-permissive conditions. Overexpression in the *hns* mutant was fully compensated by R27 in 60% of these genes, and partially compensated in an additional 14%. Therefore, there is a significant correlation between the H-NS-sensitive genes that require Hha for efficient modulation and those that are sensitive to H-NS_R27_. A significant number of those are located in HGT DNA.

## Discussion

It is well-documented and accepted that a key role of the global modulator H-NS is to silence large stretches of AT-rich HGT DNA [13]–[15],[27]. The capacity of this protein to preferentially bind to AT-rich motifs that display planar curvature probably underlies HGT DNA silencing. Efficient silencing of unwanted expression of foreign DNA appears, in turn, as a critical issue to facilitate the integration of newly acquired DNA into the host regulatory network. Nevertheless, several reports have also shown that H-NS modulates the expression of housekeeping genes, such as *proV* or *leuO*. Hence, a role for H-NS as a genome sentinel must be compatible with its function as a general gene regulator [15]. Our results open up a new perspective about how these two roles are accomplished by H-NS in enteric bacteria.

To date, plasmid-encoded H-NS-like proteins have been considered to be functionally equivalent to the corresponding chromosomally-encoded paralogues. Indeed, functional replacements and equivalent sets of interactions have been shown for plasmid- and chromosomal- H-NS-like proteins [18],[20],[22]. On the basis of this equivalence, the presence of H-NS-like proteins in plasmids has been interpreted as providing an additional source of H-NS that can compensate the depletion of resident H-NS caused by binding of the protein to AT-rich stretches of newly acquired plasmids [22]. The results reported here evidence that plasmidic and chromosomal H-NS proteins show considerable functional differences. H-NS_R27_ may contribute to reducing the fitness cost of housing HGT DNA by targeting plasmid sequences while leaving chromosomal H-NS available for its corresponding chromosomal targets, as suggested [22]. In addition, H-NS_R27_ does not influence the intrinsic regulation of H-NS- sensitive housekeeping genes, such as *rcsA*, *psiF*, *proV* or s*tpA*. The identification of a HGT-specific plasmid-encoded H-NS protein suggests that H-NS sensitive genes form two distinct genetic compartments. A relevant question is how resident chromosomal H-NS recognizes and differentially modulates genes from both compartments. We show that H-NS interacts with members of the Hha/YmoA family to specifically silence HGT but not core genome genes. Coregulation of gene expression by H-NS/Hha proteins was first evidenced for the *E. coli hly* operon [8]. Further data demonstrated that other genes in several enteric bacteria are modulated by an H-NS/Hha complex [11]–[13], rather than by H-NS alone. A recent global transcriptomic analysis has shown that, when *S.* Typhimurium SV5015 is grown in LB medium at 37°C, the set of genes sensitive to modulation by Hha and/or its paralogue YdgT is coincident with the set of H-NS-modulated genes that map in HGT DNA [26]. Remarkably, Hha/YdgT proteins appear to silence mainly HGT genes. The effect of *hha/ydgT* mutations on the transcriptome of *E. coli* strain BSN26 is very limited. Only a small number of genes is affected (our unpublished results). We interpreted these data as strain BSN26 containing a very limited amount of HGT DNA.

Genes sensitive to Hha/YdgT modulation are silenced under several *in vitro* growth conditions (i.e., low and high temperature, low and high osmolarity), and a significant number are silenced by H-NS under the same conditions. H-NS-controlled weak expression of HGT DNA [28] must be compatible with the expression of several housekeeping genes, which are readily switched on in response to specific stimuli. A good example of the latter is osmolarity-dependent modulation of *proV*, which is silenced by H-NS alone and is insensitive to Hha silencing.

We found a significant coincidence between the set of genes modulated by H-NS/Hha and those sensitive to H-NS_R27_. Plasmidic genes and chromosomal genes incorporated by HGT are prominent members of the set that are both silenced by H-NS_R27_ or require Hha to be silenced by the chromosomal form of H-NS. This observation strengthens the notion that H-NS-modulated genes can be assigned to two genetic compartments. The first includes genes encoding housekeeping functions that are modulated by chromosomal H-NS alone and are insensitive or only weakly modulated by plasmidic H-NS_R27_. In contrast, the genes belonging to the second compartment, which includes mostly horizontally acquired genes, require a helper protein of the Hha family for their complete silencing by chromosomal H-NS, and can also be modulated by plasmidic H-NS.

For many years, it was considered that H-NS did not recognize a consensus DNA sequence, but bound to AT-rich curved stretches of DNA [1]. In recent years, significant efforts have been devoted to defining high-affinity binding sites for H-NS [29],[30]. Our results suggest that H-NS-sensitive promoters fall into at least two categories. H-NS sensitive promoters may share some basic common characteristics but display differences that can be picked up by the plasmidic H-NS form or by H-NS/Hha complexes. Differential affinity of H-NS-like proteins for DNA regions targeted by H-NS has also recently been proposed [31]. The StpA paralogue binds to DNA regions similar to those bound by H-NS in *E. coli* wt cells. In contrast, only one-third of these sequences are bound by StpA in the absence of H-NS. This partial binding results in only partial StpA-mediated modulation of H-NS-sensitive genes in *hns* mutants. While the basis for such differential affinity may be the generation of either StpA-H-NS hetero- or StpA-StpA homodimers, in the example reported here structural differences between H-NS and H-NS_R27_ might account for the differential affinity of these two proteins for some promoter regions. Sequence conservation between H-NS_R27_ and H-NS in the N- and C-terminal domains was very high; however, significant differences between H-NS and H-NS_R27_ were located in the linker domain (53% of the positions were different) (data not shown). The linker domain is predicted to be partially unstructured in the isolated protein and is associated with protein oligomerization although it has also been implicated in the modulation of DNA binding [32].

While H-NS-like proteins are widely distributed within γ-proteobacteria, both Hha-like proteins and the presence of H-NS-like proteins in conjugative plasmids appear to be an evolutionary trait of members of the *Enterobacteriaceae* [33]. Members of this family display the ability to use the H-NS protein to silence HGT regions with the help of co-repressors of the Hha family of proteins. Specialized plasmidic H-NS-like molecules also have the capacity to modulate HGT DNA, but have evolved not to interfere with core genes. These features should facilitate the incorporation of HGT DNA, leading to more complex genomes with increased capability to adapt to new environments. This adaptive capacity may also explain why several enterobacterial representatives, such as virulent *E. coli* strains, have become such successful pathogens [34],[35].

## Materials and Methods

### Plasmids and strains

Bacterial strains and plasmids used in this work are described in Text S1, Table S4, and Table S5.

### β-galactosidase assay

Levels of β-galactosidase activity were assayed by standard techniques, using the CHCl_3_-sodium dodecyl sulfate permeabilization procedure.

### Overexpression of proteins by the T7 RNA polymerase system and purification of His-Tagged proteins

*E. coli* BL21 (DE3) Δ*hns* strain was used as host induction of H-NS-like R27 protein expression. Plasmid pETHNSR27his was introduced by transformation into this strain. One-liter culture was grown to an OD_600_ of 0.3, and at this point IPTG was added to 0.5 mM. Incubation at 37°C continued for 2 h. Cells were pelleted by centrifugation and resuspended in 20 mL buffer A (20 mM Hepes pH 7.9, 100 mM KCl, 5 mM MgCl_2_, 20 mM imidazole). The cells were lysed by three passages through a French press at 1000 p.s.i. Plasmid pETHNSHIS was used to overexpress His-tagged H-NS protein as described previously [36]. His-tagged proteins were purified from the soluble extract with Ni^2+^-NTA agarose (Qiagen).

### Band-shift assays

Electrophoretic band-shift assays were performed as described previously [5]. DNA fragments corresponding to the promoter region of *proV*, *hilA* or *rcsA* genes were amplified by PCR using primers hilA-BS-5/hilA-BS-3, proU-BS-5/proU-BS-3 and rcsA-BS-3/rcsA-BS-5 respectively (Table S5).

### RNA isolation, microarray procedure, and data analysis

Transcriptomic analyses were performed on a DNA microarray engineered by the Salgenomics consortium of research groups. The Salgenomics microarray contained 6,119 probes (including open reading frames, RNA genes and intergenic regions) from the genome sequence of *S. enterica* serovar Typhimurium SL1344 and was developed using sequences from the Welcome Trust Sanger Institute. RNA extraction, retrotranscription, labeling, hybridization, microarray scanning, and data analysis were performed as described elsewhere [37].

## Supporting Information

Figure S1 Expression of β-galactosidase from *lac* fusions to *proV* in strains SV5015 (wt), SV5015AV (*hnsM*) and SV5015H (*hha*).
(0.89 MB TIF)

Table S1 Genes induced more than 2-fold (M≥1) in SV5015AV with respect to SV5015 with a p value less than 0.1, and the corresponding values in SV5015AV (R27) vs SV5015. Significative HGT genes (SPIs, pSLT) have been highlighted in grey. Significative core genome genes have been highlighted in black.
(0.12 MB XLS)

Table S2 Genes repressed more than 2-fold (M≤−1) in SV5015AV with respect to SV5015 with a p value less than 0.1, and the corresponding values in SV5015AV (R27) vs SV5015. Significative repressed genes (cell motility) have been highlighted in grey. Significative core genome genes have been highlighted in black.
(0.16 MB XLS)

Table S3 Genes induced more than 2-fold (M≥1) in SV5015HY, SV5015AV, and SV5015AV (R27) with respect to SV5015 with a p value less than 0.1.
(0.07 MB XLS)

Table S4 Bacterial strains and plasmids used in this study.
(0.06 MB DOC)

Table S5 Oligonucleotides used in this work.
(0.05 MB DOC)

Text S1 Bacterial strains and growth conditions.
(0.03 MB DOC)

## References

[1] Dorman CJ (2004). H-NS: a universal regulator for a dynamic genome.. Nat Rew Microbiol.

[2] Rimsky S (2004). Structure of the histone-like protein H-NS and its role in regulation and genome superstructure.. Curr Opin Microbiol.

[3] Falconi M, Colonna B, Prosseda G, Micheli G, Gualerzi CO (1998). Thermoregulation of *Shigella* and *Escherichia coli* EIEC pathogenicity. A temperature-dependent structural transition of DNA modulates accesibility of the *virF* promoter to transcriptional repressor H-NS.. EMBO J.

[4] Dorman CJ, Deighan P (2003). Regulation of gene expression by histone-like proteins in bacteria.. Curr Opin Genet Dev.

[5] Madrid C (2002). Temperature- and H-NS-dependent regulation of a plasmid-encoded virulence operon expressing *Escherichia coli* hemolysin.. J Bacteriol.

[6] Stella S, Spurio R, Falconi M, Pon CL, Gualerzi CO (2005). Nature and mechanism of the *in vivo* oligomerization of nucleoid protein H-NS.. EMBO J.

[7] Madrid C, Balsalobre C, García J, Juárez A (2007). The novel Hha/YmoA family of nucleoid-associated proteins: use of structural mimicry to modulate the activity of the H-NS family of proteins.. Mol Microbiol.

[8] Nieto JM (2000). Expression of the hemolysin operon in *Escherichia coli* is modulated by a nucleoid-protein complex that includes the proteins Hha and H-NS.. Mol Gen Genet.

[9] Olekhnovich IN, Kadner RJ (2006). Crucial roles of both flanking sequences in silencing of the *hilA* promoter in *Salmonella enterica*.. J Mol Bio.

[10] Ellison DV, Miller VL (2006). H-NS represses *inv* transcription in *Yersinia enterocolitica* through competition with RovA and interaction with YmoA.. J Bacteriol.

[11] Silphaduang U, Mascarenhas M, Karmali M, Coombes BK (2007). Repression of intracellular virulence factors in *Salmonella* by the Hha and YdgT nucleoid-associated proteins.. J Bacteriol.

[12] Ono S (2005). H-NS is part of a thermally controlled mechanism for bacterial gene regulation.. Biochemical J.

[13] Navarre WW (2006). Selective silencing of foreign DNA with low GC content by the H-NS protein in *Salmonella*.. Science.

[14] Lucchini S, Rowley G, Goldberg MD, Hurd D, Harrison M (2006). H-NS mediates the silencing of laterally acquired genes in bacteria.. PloS Pathog.

[15] Dorman CJ (2007). H-NS, the genome sentinel.. Nat Rew Microbiol.

[16] Wain J (2003). Molecular analysis of incHI1 antimicrobial resistance plasmids from *Salmonella* serovar Typhi strains associated with typhoid fever.. Antimicrobial Ag Chemoter.

[17] Sherburne CK (2000). The complete DNA sequence and analysis of R27, a large IncHI plasmid from *Salmonella typhi* that is temperature sensitive for transfer.. Nucleic Acids Res.

[18] Forns N, Baños RC, Balsalobre C, Juárez A, Madrid C (2005). Temperature-dependent conjugative transfer of R27: role of chromosome- and plasmid-encoded Hha and H-NS proteins.. J Bacteriol.

[19] Beloin C, Deighan P, Doyle M, Dorman CJ (2003). *Shigella flexneri* 2a strain 2457T expresses three members of the H-NS-like protein family: characterization of the Sfh protein.. Mol Gen Genomics.

[20] Deighan P, Beloin C, Dorman CJ (2003). Three-way interactions among the Sfh, StpA and H-NS nucleoid-structuring proteins of *Shigella flexneri* 2a strain 2457T.. Mol Microbiol.

[21] Wei J (2003). Complete genome sequence and comparative genomics of *Shigella flexneri* serotype 2a strain 2457T.. Infect Immun.

[22] Doyle M (2007). An H-NS-like stealth protein aids horizontal DNA transmission in bacteria.. Science.

[23] Higgins CF (1988). A physiological role for DNA supercoiling in the osmotic regulation of gene expression in *S. typhimurium* and *E. coli*.. Cell.

[24] Owen-Hughes T (1992). The chromatin-associated protein H-NS interacts with curved DNA to influence DNA topology and gene expression.. Cell.

[25] Olekhnovich IR, Kadner RJ (2006). Crucial roles of both flanking sequences in silencing of the hilA promoter in *Salmonella enterica*.. J Mol Biol.

[26] Vivero A (2008). Modulation of horizontally acquired genes by the Hha-YdgT proteins in *Salmonella enterica* serovar Typhimurium.. J Bacteriol.

[27] Castang S, McManus HR, Turner KH, Dove SL (2008). H-NS family members function coordinately in an opportunistic pathogen.. Proc Natl Acad Sci USA.

[28] Taoka T (2004). Only a small subset of the horizontally transferred chromosomal genes in *Escherichia coli* are translated into proteins.. Mol Cel Proteomics.

[29] Bouffartigues E, Buckle M, Badaut C, Travers A, Rimsky S (2007). H-NS cooperative binding to high-affinity sites in a regulatory element results in transcriptional silencing.. Nat Struct Mol Biol.

[30] Lang B (2007). High-affinity DNA binding sites for H-NS provide a molecular basis for selective silencing within proteobacterial genomes.. Nucleic Acids Res.

[31] Uyar E (2009). Differential binding profiles of StpA in wild-type and *hns* mutant cells: a comparative analysis of cooperative partners by chromatin immunoprecipitation-microarray analysis.. J Bacteriol.

[32] Shindo H (1999). Identification of the DNA binding surface of H-NS protein from *Escherichia coli* by heteronuclear NMR spectroscopy.. FEBS Lett.

[33] Madrid C, García J, Pons M, Juárez A (2007). Molecular evolution of the H-NS protein: interaction with Hha-like proteins is restricted to enterobacteriaceae.. J Bacteriol.

[34] Wirth T (2006). Sex an virulence in *Escherichia coli*: an evolutionary perspective.. Mol Microbiol.

[35] Pigtout JD, Laupland KB (2008). Extended-spectrum beta-lactamase-producing Enterobacteriaceae: an emerging public-health concern.. Lancet Infect Dis.

[36] Nieto JM (2002). Evidence for direct protein-protein interaction between members of the enterobacterial Hha/YmoA and H-NS families of proteins.. J Bacteriol.

[37] Mariscotti JF, García-del Portillo F (2009). Genome expression analyses revealing the modulation of the *Salmonella* Rcs regulon by the attenuator IgaA.. J Bacteriol.

