# Supplementary figures and images for: Differential Regulation of Horizontally Acquired and Core Genome Genes by the Bacterial Modulator H-NS
Source: PLoS Genet. 2009 Jun 12;5(6):e1000513. doi: 10.1371/journal.pgen.1000513 (PMC2686267; doi:10.1371/journal.pgen.1000513)

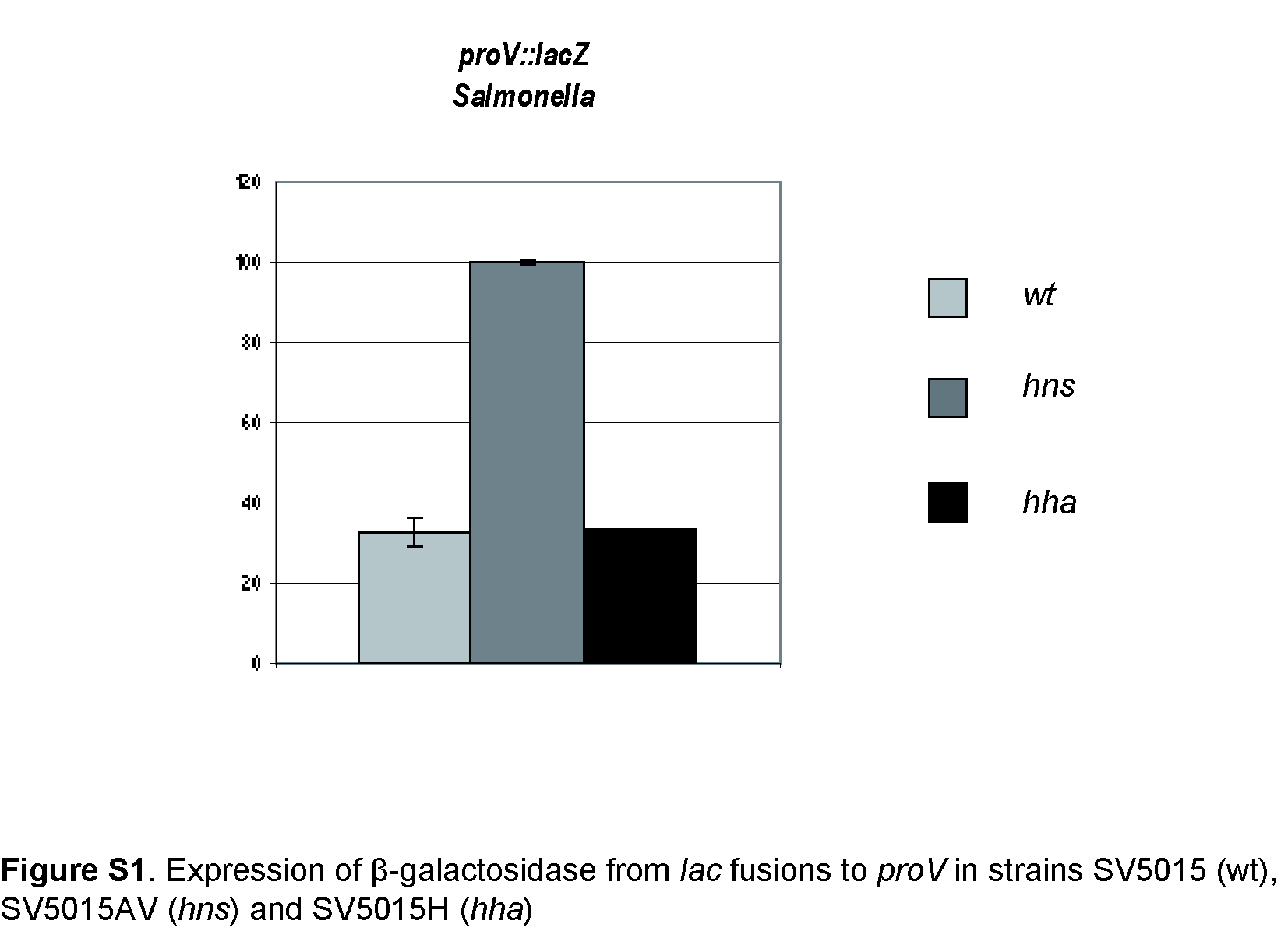

Supplement: Figure S1 — Expression of β-galactosidase from lac fusions to proV in strains SV5015 (wt), SV5015AV (hnsM) and SV5015H (hha). (0.89 MB TIF) [file pgen.1000513.s001.tif]
