# Supplementary material for: Differential Regulation of Horizontally Acquired and Core Genome Genes by the Bacterial Modulator H-NS
Source: PLoS Genet. 2009 Jun 12;5(6):e1000513. doi: 10.1371/journal.pgen.1000513 (PMC2686267; doi:10.1371/journal.pgen.1000513)
Supplement: Table S4 — Bacterial strains and plasmids used in this study. (0.06 MB DOC) [file pgen.1000513.s005.doc]

**Table S4**. Bacterial strains and plasmids used in this study.

| **Strain/plasmid** | **Relevant characteristics** | **Reference / Source** |
| --- | --- | --- |
| ***S*. Typhimuium** |  |  |
| SV5015 | SL1344 His^+^ | J. Casadesús |
| SV5015H | SV5015 Δ*hha*::Cm | 1 |
| SV5015HY | SV5015 Δ*hha*::Cm Δ*ydgt*::Km | 1 |
| SV5015AV | SV5015 Δ*hns*::Km | This work |
| SV5015UB1 | SV5015 Ф*proV*-‘*lacZY* | This work |
| SV5015UB2 | SV5015 Ф*hilA-‘lacZY* | M. Hüttener |
| SV5015UB3 | SV5015 Ф*rcsA-‘lacZY* | This work |
| ***E. coli*** |  |  |
| BL21 (DE3) | *hsdS gal* (*c*I*ts*857 *ind-1 Sam*7 *nin-5 lacUV5*-T*7* gene 1) | 2 |
| BL21 (DE3) ∆*hns* | BL21 (DE3) ∆*hns::*Km | 3 |
| 5K | F^-^ *hsdR hsdM thr thi leu lacZ* | 4 |
| 5K *hns* | 5K *hns*::Ap^r^ | 5 |
| 5KUB1 | 5K Ф*proV*-*lacZ* | This work |
| **Plasmids** |  |  |
| R27 | IncHI1, Tc^r^ | 6 |
| R27Δhns | R27 Δ*hns* | 5 |
| pHly152 | *hlyR, hlyC, hlyA, hlyB, hlyC* | 7 |
| pHlyA::lacZ | pHly152 *hlyA::lacZ* | J. Fernández-Vázquez |
| pCP20 | FLP helper plasmid, Ap^r^ Cm^r^ | 8 |
| pKD46 | Red helper plasmid, Ap^r^ | 9 |
| pKG136 | Km^r^ FRT lacZY^+^ t_his_ oriR6K | J.M. Slauch |
| pKG137 | Km^r^ FRT lacZY^+^ t_his_ oriR6K | J.M. Slauch |
| pKD3 | Template plasmid, Cm^r^ | 9 |
| pKD4 | Template plasmid, Km^r^ | 9 |
| pETHNSR27His | pET22b + *hns-* R27 His-Tag, Ap^r^ | This work |
| pETHNSHis | pET3b + *hns* His-Tag, Ap^r^ | 10 |
| pLysE | Cm^r^ | 2 |

**References**

1. Vivero A, Baños RC, Mariscotti, JF, García-del Portillo F et al. (2008) [Modulation of horizontally acquired genes by the Hha-YdgT proteins in *Salmonella* *enterica* serovar Typhimurium.](http://www.ncbi.nlm.nih.gov/pubmed/18039769?ordinalpos=1&itool=EntrezSystem2.PEntrez.Pubmed.Pubmed_ResultsPanel.Pubmed_DefaultReportPanel.Pubmed_RVDocSum) J Bacteriol 190: 1152-1156.

2. Studier FW, Rosenberg AH, Dunn JJ, Dubendorff JW (1990) Use of T7 RNA polymerase to direct expression of cloned genes. Methods Enzymol 185:60-89

3. Zhang A, Rimsky S, Reaban ME, Buc H, Belfort M (1996) *Escherichia coli* protein analogs StpA and H-NS: regulatory loops, similar and disparate effects on nucleic acid dynamics. EMBO J 15:1340-1349.

4. Juárez A, Hughes C, Vogel M, Goebel W (1984) Expression and regulation of the plasmid-encoded hemolysin determinant of *Escherichia coli*. Mol Gen Genet 197:196-203.

5. Forns N, Baños RC, Balsalobre C, Juárez A, Madrid C (2005) Temperature-dependent conjugative transfer of R27: role of chromosome- and plasmid-encoded Hha and H-NS proteins.J Bacteriol 187:3950-3959.

6. Grindley NDF, Grindley JN, Anderson ES (1972) R factor compatibility groups Mol Gen Genet 119:287-297.

7. Noegel A, Rdest U, Goebel W (1981) Determinations of the functions of hemolytic plasmid pHly152 of *Escherichia coli*. J Bacteriol 145:233-247.

8. Cherepanov PP, Wackernagel W (1995) Gene disruption in *Escherichia* *coli*: TcR and KmR cassettes with the option of Flp-catalyzed excision of the antibiotic-resistance determinant. Gene 1995 158:9-14.

9. Datsenko KA, Wanner BL (2000) One-step inactivation of chromosomal genes in *Escherichia coli* K-12 using PCR products. Proc Natl Acad Sci USA 97:6640-6645.

10. Nieto JM, Madrid C, Miquelay E, Parra JL, Rodríguez S et al. (2002) Evidence for direct protein-protein interaction between members of the enterobacterial Hha/YmoA and H-NS families of proteins. J Bacteriol 184:629-635.
