# Supplementary material for: Differential Regulation of Horizontally Acquired and Core Genome Genes by the Bacterial Modulator H-NS
Source: PLoS Genet. 2009 Jun 12;5(6):e1000513. doi: 10.1371/journal.pgen.1000513 (PMC2686267; doi:10.1371/journal.pgen.1000513)
Supplement: Table S5 — Oligonucleotides used in this work. (0.05 MB DOC) [file pgen.1000513.s006.doc]

Table S5. Oligonucleotides used in this work

| **Name** | **Sequence 5’🡪3’** |
| --- | --- |
| hilAP1 | ATGCCACATTTTAATCCTGTTCCTGTATCGAATAAAAAATTCGTGTAGGCTGGAGCTGCTTC |
| hilAP2 | TTACCGTAATTTAATCAAGCGGGGATCCTGTTTCCATCTTTTCATATGAATATCCTCCTTAGT |
| hilaP1Up | ATTATCATGCCACATTTTAATCC |
| hilAP2Down | TTACCGTAATTTAATCAAGCG |
| ProUP1B | CGCTGAGCTTCGCGAGGTGCGCAGGAAAAAGATTGCGATGGTGTAGGCTGGAGCTGCTTC |
| ProUP2B | CGACGTGAGAGAGCAACTCGCTGAGTGGGGTTTGCGCATCCATATGAATATCCTCCTTAGT |
| ProU-1 | AGGTACTGATTGACGGCGTT |
| ProU-2 | TGTTCTTCATCGACAACCGG |
| hilA-BS-5 | AGCCAGCCTGAGGATGATAC |
| hilA-BS-3 | GCTTCGAGCAGGATGACCAG |
| proU-BS-5 | AGGGAGTCAAATCGCGCAAA |
| proU-BS-3 | GGGTTCAATCAGGCGATTGA |
| rcsA-P1 | TATGGATTTGTGCAGTTACACCCGGCTAGGGTTAAGCGGGGTGTAGGCTGGAGCTGCTTC |
| rcsA-P2 | TTAACAAAAATGCCGTTAGTGACGTTATCTGTCAGCCGTACATATGAATATCCTCCTTAGT |
| KT | CGGCCACAGTCGATGAATCC |
| rcsA-BS-5 | AGGATATATCCACTGCCCCT |
| rcsA-BS-3 | TTCGTCAACGGTTTCGATGT |
| HNSP1 | CCACCCCAATATAAGTTTGAGATTACTACAATGAGCGAAGTGTAGGCTGGAGCTGCTTC |
| HNSP2 | ATCCAGGAAGTAAATTATACCTTGATCAGGAAATCTTCCAGCATATGAATATCCTCCTTAGT |
| HNS-3 | CCACCCCAATATAAGTTTGAG |
| HNS-4 | CGGCGGGATTTTAAGCATCCA |
| ORF164NDE | GGAATTCCATATGTCCGAAGCACTCAAATC |
| ORF164XHO | CCGCTCGAGGAAATCATCCAGTTTTTT |
| HlyA-P1 | ATCATATCCATTTTCAAAGTAATTTTTGCCGTGTTTTGTGTAGGCTGGAGCTGCTTC |
| HlyA-P2 | CAAATTAAAAGCACACTGCAGTCTGCAAAGCAATCCGCATATGAATATCCTCCTTAGT |
| GUNO | GTATCTTTGTTTGCAATAAATGTA |
| CAT-C2 | GATCTTCCGTCACAGGTAGG |
