# Supplementary material for: Differential Regulation of Horizontally Acquired and Core Genome Genes by the Bacterial Modulator H-NS
Source: PLoS Genet. 2009 Jun 12;5(6):e1000513. doi: 10.1371/journal.pgen.1000513 (PMC2686267; doi:10.1371/journal.pgen.1000513)
Supplement: Text S1 — Bacterial strains and growth conditions. (0.03 MB DOC) [file pgen.1000513.s007.doc]

**Text S1**

**Bacterial strains and growth conditions**.

Bacterial strains and plasmids used in this work are listed in Table S4. The strains were routinely grown in Luria-Bertani (LB) medium (10 g NaCl, 10 g tryptone and 5 g yeast extract per liter). For mating experiments, cultures of donor and recipient strains were grown in Penassay broth (1.5 g meat extract, 1.5 g yeast extract, 5 g peptone, 1 g glucose, 3.5 g NaCl, 1.32 g KH_2_PO_4_, 4.82 g K_2_HPO_4_ · 3H_2_O per liter). For β-galactosidase assays cells were grown in LB medium at 37ºC to late exponential phase of growth.The antibiotics used were kanamycin (50 µg/ml), ampicillin (100 µg/ml), chloramphenicol (20 µg/ml) and tetracycline (15 µg/ml).

*Salmonella* Typhimurium SV5015 is a His+ derivative of SL1344 strain. Chromosomal deletion of the *hns* gene was done by the λ Red recombinant method as described by Datsenko and Wanner [1]. The antibiotic resistance of plasmid pKD4 (kanamicin) was amplified using primers HNSP1 and HNSP2. The resulting PCR product was purified and electroporated into SV5015 strain. Deletion of *hns* was verified by PCR using primers HNS-3 and HNS-4.

To construct transcriptional *lac* fusions in *hilA,* *proV* and *rcsA* genes in *Salmonella*, and *hly::lacZ* in *E.coli*, the genes were first disrupted in strains SV5015 and 5K (pHly152) respectively, using the procedure of Datsenko and Wanner [1]. The oligonucleotides used for these constructions were hilAP1/hilAP2, ProUP1B/ProUP2B, rcsAP1/rcsAP2 and HlyA-P1/HlyA-P2, which amplified the antibiotic resistance of plasmid pKD3 (cloramphenicol) or pKD4 (kanamycin) with extensions corresponding to sequences of *hilA*, *proV*, *rcsA* and *hlyA* respectively. After verification of the predicted deletions using primers hilaP1Up/hilAP2Down, ProU-1/ProU-2, rcsA-BS-5/KT and GUNO/CAT-C2, FRT sites generated by excision of the antibiotic cassettes were used to integrate plasmid pKG136 or pKG137 [2], thereby generating trancriptional *lac* fusions. Transfer *lac* fusions between *Salmonella* strains was carried out by P22 HT transduction [3]. Transfer *lac* fusions between *E.coli* strains was carried out by P1vir transduction [4].Transcriptional fusion to *proV* gene in *E.coli* 5K was done by P1vir transduction of *proU::lacZ* transcriptional fusion from *E.coli* GM37 [5].

To construct plasmid pETHNSR27His, the *hns* gene from R27 was amplified by PCR using primers ORF164NDE, which adds an *Nde*I site to the sequence encoding the N-terminus of H-NS-like R27 protein, and ORF164XHO which adds a *Xho*I site before the last codon at the C-terminus of H-NS-like R27 protein. The *Nde*I-*Xho*I PCR fragment was cloned into pET22b digested with the same restriction enzymes.

R27 or R27*hns* plasmids were transferred to different recipient strains as described previously [6].

**References**

1. Datsenko KA, Wanner BL (2000) One-step inactivation of chromosomal genes in *Escherichia coli* K-12 using PCR products. Proc Natl Acad Sci USA 97:6640-6645.

2. Ellermeier CD, Janakiraman A, Slauch JM (2002) Construction of targeted single copy lac fusions using lambda Red and FLP-mediated site-specific recombination in bacteria. Gene 290:153-161.

3. Schmieger H (1972) Phage P22-mutants with increased or decreased transduction abilities. Mol Gen Genet 119:75-88.

4. Miller JH (1992) A short course in bacterial genetics. A laboratory manual and handbook for *Escherichia coli* and related bacteria. Cold Spring Harbor Laboratory Press, Cold Srping Harbor, N.Y.

5. Hulton CS, Seirafi A, Hinton JC, Sidebotham JM et al (1990) Histone-like protein H1 (H-NS), DNA supercoiling, and gene expression in bacteria. Cell 63:631-642.

6. Taylor DE, Levine JG (1980) Studies of temperature-sensitive transfer and maintenance of H incompatibility group plasmids. J Gen Microbiol 116:475-484.
